# *p*-Sulfonic Acid Calix[4]arene as an Efficient Catalyst for One-Pot Synthesis of Pharmaceutically Significant Coumarin Derivatives under Solvent-Free Condition

**DOI:** 10.1155/2015/738202

**Published:** 2015-12-20

**Authors:** Hamed Tashakkorian, Moslem Mansour Lakouraj, Mona Rouhi

**Affiliations:** ^1^Cellular and Molecular Biology Research Center (CMBRC), Health Research Institute, Babol University of Medical Sciences, Babol 47176 41367, Iran; ^2^Department of Organic Chemistry, Faculty of Chemistry, University of Mazandaran, Babolsar 47416 95447, Iran

## Abstract

One-pot and efficient protocol for preparation of some potent pharmaceutically valuable coumarin derivatives under solvent-free condition via direct coupling using biologically nontoxic organocatalyst, calix[4]arene tetrasulfonic acid (CSA), was introduced. Calix[4]arene sulfonic acid has been incorporated lately as a magnificent and recyclable organocatalyst for the synthesis of some organic compounds. Nontoxicity, solvent-free conditions, good-to-excellent yields for pharmaceutically significant structures, and especially ease of catalyst recovery make this procedure valuable and environmentally benign.

## 1. Introduction

Finding novel synthetic procedures for a variety of attractive compounds that can be considered as pharmaceutics has been investigated over last decades. The introduction of new methodologies in recent years based on environmentally friendly conditions using efficient and reusable catalyst as well as solvent-free procedures has gained significant attentions among the researchers. Among the thoroughly investigated organic structures, coumarins and their derivatives were highly regarded due to their vast applications in pharmaceutical industries. Coumarins, also recognized as benzopyrones, have revealed characteristics of these potent heterocyclic compounds and proved to feature significant biological activities including antimicrobial, antiviral, antitumor, and antioxidant properties. For instance, monoamine oxidase inhibitors (MAO) have antidiabetic activity [1], antiallergic activity [2], anabolic antioxidant, and hepatoprotective activities [3, 4]. These valuable structures, natural or synthetic ones, due to their mentioned characteristics are multipurpose compounds and play variety of roles in our lives. On the other hand, they are being applied to agrochemical [5], food, and cosmetic industries as additives to serve as optical brighteners [6, 7], anticoagulants [8], and laser dyes [9, 10] and they have significant therapeutic roles in pharmaceuticals [11] and treatment of cancer [12]. Experimental surveys exhibit the noteworthy chemotherapeutics activities of these organic structures and they hence have been employed as inhibitors in growth of diverse human tumour cell lines [13].

Their known functionalized families have shown variety of antioxidant [14, 15], anti-HIV, anticoagulant, hypotensive, and spasmolytic activities [16–18]. Besides, some potential antibiotic characteristics have been reported for coumarin derivatives such as chartesium, coumermycin, and novobiocin [19]. These valuable compounds also have proved their efficiency as biologically active structures in the formulation of some medicines for a long time [20, 21]. As being natural molecules, coumarins are among the phytochemical compounds that exist in high concentration in tonka bean, apricots, cherries, cinnamon, mullein, strawberries, and some other natural products. However, due to their valuable and impressive characteristics which resulted in being employed in the wide range of products, they became desirable molecules for researchers to find the least harmful and the most efficient preparative procedures. In synthetic chemistry some methods were considered as famous and pioneer methods for preparation of these organic compounds. The first reported procedure for preparation of coumarins was Perkin reaction that was suggested in 1868 [22]. However, Perkin reaction is efficient and suitable just for synthesis of simple coumarins but some alternative methods were developed during the years including Pechmann [23], Knoevenagel [24, 25], Reformatsky [26], Kostanecki-Robinson [27], and Wittig reactions [28, 29]. Although these categories of well known reactions give coumarins in acceptable yields, usually they need severe conditions such as high temperatures, dangerous solvents, long reaction time, and producing byproducts. Excess amounts of harsh acidic activating agents such as POCl_3_, P_2_O_5_, and polyphosphoric acid besides quantitative amount of Lewis acids or sulfuric or sulfonic acids add to their usage drawbacks due to possible severe difficulties such as corrosion problem.

So far, different catalyses have been raised to overcome the mentioned drawbacks in the last decade [30–32]. One of the motivating macrocyclic structures with great capacity in different area and that attracted much interest especially in biochemistry is calixarene. Among the interesting compounds derived from this scaffold, sulfonate calixarene has aroused much interest in biopharmaceutical applications [33], due to its promising capabilities in incapsulating; calixarenes increase not only solubility but also bioavailability of valuable pharmaceutical drugs such as nifedipine [34, 35], furosemide [36], niclosamide [37], carbamazepine [38], and topotecan for improving solubility in chemotherapy by host-guest complexes [39]. Surprisingly, medical experiments either of in vivo studies or at the cellular level indicate that the calixarenes have no activity in the Ames test [37]. Moreover, no acute toxicity for the sulfonated calixarenes is reported when specified amounts were injected in mice for in vivo studies. Number of previous organized studies revealed that these multipurpose compounds are not cytotoxic [40]. Calixarenes have very favorable physicochemical properties that are similar to other useful pharmaceutical excipients such as cyclodextrins [41]. Besides the biocompatibility of sulfonated calixarenes, investigation on catalytic activity of this motif in the synthesis of pharmaceutical compounds seems to be interesting. In this regard and in continuation of our recent studies on the development of convenient, effective, and safe protocol in organic and pharmaceutical synthesis [42–45], herein we represent calix[4]arene tetrasulfonic acid, as an efficient and environmentally friendly organocatalyst for the preparation of coumarin derivatives under solvent-free conditions.

## 2. Experimental

### 2.1. Materials and Instruments

**Scheme 1:**
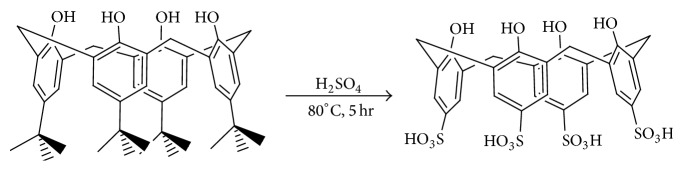
Sulfonation of calix[4]arene using Shinkai method.

**Figure 1:**
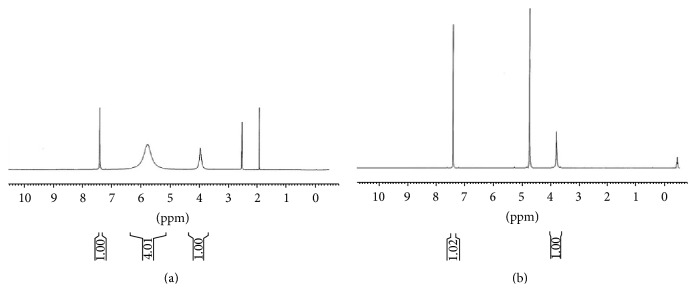
^1^H NMR spectra of calix[4]arene sulfonic acid in (a) DMSO-*d*_*6*_ and (b) D_2_O.

**Figure 2:**
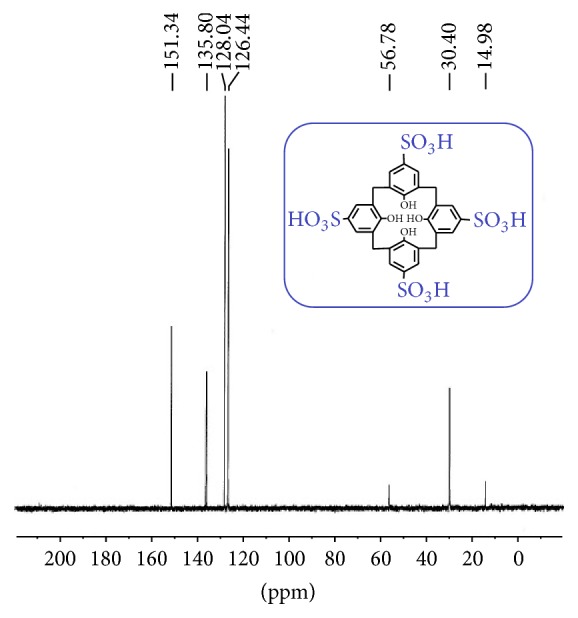
^13^C NMR spectrum of calix[4]arene sulfonic acid in D_2_O.

The fine chemicals including* p*-tert-butylphenol, formaldehyde solution (37%), diphenyl ether, ethylacetate, methanol, and sodium hydroxide were purchased from Merck (Schuchardt, Germany). Ethyl acetoacetate, resorcinol, bisphenols, salicylic acid, 2,5-dihydroxy salicylic acid, phosphorus oxychloride, and silicagel were obtained from Fluka (Switzerland). Parent tert-butylcalixarene was synthesized according to Gutsche procedure published in [51]. Then it was detertiobutylated and sulfonated simultaneously by Shinkai method using concentrated sulfuric acid (Scheme 1) [52]. After further purification described in the literature, the obtained product was applied as a proficient acidic organocatalysis (Figures 1 and 2). Melting points were determined with an Electrothermal 9100 Melting Point Apparatus. IR and ^1^H NMR spectra were recorded, respectively, on Bruker FTIR Spectrometer and Bruker Avance III 400 MHz NMR Spectrometer. GC-MS analyses were carried out on Shimadzu GC 17A gas chromatograph coupled with MS-QP 5000 Shimadzu Mass Spectrometer (Tokyo, Japan). Elemental analyses were performed by CHN Rapid Heraeus Elemental Analyzer (Wellesley, MA).

### 2.2. General Procedure for Preparation of Coumarins

**Figure 3:**
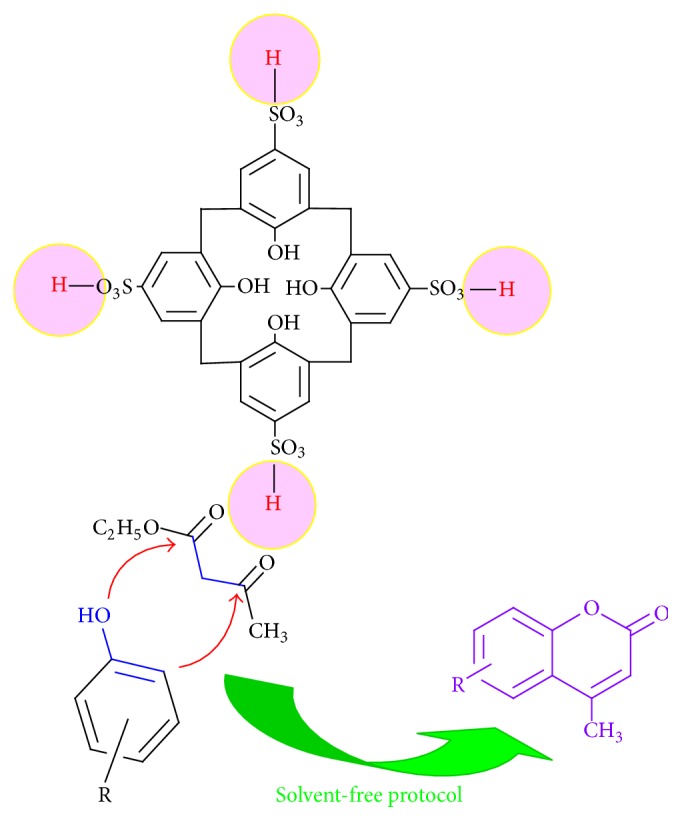
Schematic preparation of coumarin.

A specified amount of the catalyst, CSA (45 mg, 0.06 mmol), was added to the mixture of ethyl acetoacetate (130 mg, 1 mmol) and phenol derivatives (1 mmol). Then, the The flask was placed in an oil bath and temperature was adjusted to 90°C. After completion of the reaction which was monitored by TLC, it was cooled down to room temperature and then poured onto the crushed ice. After half an hour, reaction mixture was extracted with chloroform three times (3 × 25 mL). Subsequently, the combined organic phase was washed with saturated aqueous sodium bicarbonate, brine solution, and water, respectively. Then organic solution was dried using magnesium sulfate and the crude product was obtained using rotary evaporator. For further purification flash column chromatography was performed using petroleum ether/ethylacetate, 90 : 10 (Figure 3).

### 2.3. Coumarin Derivatives Characterization

*4-Methyl-2H-chromen-2-one (* ***1a***). This compound was obtained (24 mg, 15%) and characterized according to the described procedure from Ethyl acetoacetate (130 mg, 1 mmol) and resorcinol (110 mg, 1 mmol).

IR (*υ*_max_/cm^−1^): 1064, 1238, 1543, 1705, 3020.

^1^H NMR (400 MHz, CDCl_3_), (*δ*: ppm): *δ* 2.42 (d, 3H), 6.32 (q, 1H), 7.15–7.42 (m, 3H), 7.48 (d, *J* = 6.0 Hz, 1H).

^13^C NMR (100 MHz, CDCl_3_), (*δ*: ppm): 19.1, 116, 117.9, 122, 124.1, 124.6. 132.6, 153, 154.5, 161.6, ESI-MS *m*/*z*: 160; Anal. Calcd for C_10_H_8_O_2_: C: 75.00, H: 5.00. Found: C: 75.23, H: 5.18.

*7-Hydroxy-4-methyl-2H-chromen-2-one (* ***2a***). This compound was prepared (167 mg, 95%) and characterized according to the described procedure from Ethyl acetoacetate (130 mg, 1 mmol) and resorcinol (110 mg, 1 mmol).

IR (*υ*_max_/cm^−1^): 1060, 1227, 1590, 1680, 3150.

^1^H NMR (400 MHz, CDCl_3_), (*δ*: ppm): 2.35 (d, 3H), 6.11 (q, 1H), 6.69 (d, *J* = 2.4 Hz, 1H), 6.78 (dd, *J* = 8.8 Hz, 1H), 7.56 (d, *J* = 8.8 Hz, 1H), 10.52 (s, 1H).

^13^C NMR (100 MHz, CDCl_3_), (*δ*: ppm): 18.56, 102.62, 110.70, 112.47, 113.29, 127.05, 153.97, 155.28, 160.74, 161.6. ESI-MS *m*/*z*: 176; Anal. Calcd for C_10_H_8_O_3_: C: 68.18, H: 4.54. Found: C: 68.45, H: 4.76.

*6-Hydroxy-4-methyl-2H-chromen-2-one (* ***3a***). This compound was obtained (132 mg, 75%) and identified according to the described procedure from Ethyl acetoacetate (130 mg, 1 mmol) and hydroquinone (110 mg, 1 mmol).

IR (*υ*_max_/cm^−1^): 1055, 1225, 1565, 1693, 3010, 3412.

^1^H NMR (400 MHz, CDCl_3_), (*δ*: ppm): 2.42 (d, 3H), 6.33 (q, 1H), 6.73 (d, *J* = 8.4 Hz, 1H), 6.81 (d, *J* = 8.4 Hz, 1H), 7.28 (s, 1H).

^13^C NMR (100 MHz, CDCl_3_), (*δ*: ppm): 18.38, 109.40, 114.40, 117.50, 120.65, 121.54, 147.10, 153.93, 155.10, 159.70. ESI-MS *m*/*z*: 176; Anal. Calcd for C_10_H_8_O_3_: C: 68.18, H: 4.54. Found: C: 68.36, H: 4.88.

*4,7-Dimethyl-2H-chromen-2-one (* ***4a***). This compound was prepared (87 mg, 50%) and identified according to the mentioned procedure from Ethyl acetoacetate (130 mg, 1 mmol) and m-cresol (108 mg, 1 mmol).

IR (*υ*_max_/cm^−1^): 1070, 1146, 1212, 1248, 1378, 1579, 1636, 1704, 2920, 2970.

^1^H NMR (400 MHz, CDCl_3_), *δ* (ppm): 2.10 (s, 3H), 2.31 (d, 3H), 4.17 (s, 1H), 6.71–7.29 (m, 3H).

^13^C NMR (100 MHz, CDCl_3_), *δ* (ppm): 18.04, 24.17, 110.70, 117.43, 118.20, 124.10, 126.50, 144.50, 152.74, 153.92, 162.1. ESI-MS *m*/*z*: 174; Anal. Calcd for C_11_H_10_O_2_: C: 75.86, H: 5.74. Found: C: 75.52, H: 5.49.

*7-Methoxy-4-methyl-2H-chromen-2-one (* ***5a***). This compound was obtained (171 mg, 90%) and charectarized according to the mentioned procedure from Ethyl acetoacetate (130 mg, 1 mmol) and m-methoxy phenol (124 mg, 1 mmol).

IR (*υ*_max_/cm^−1^): 1068, 1284, 1510, 1725, 2927, 3070.

^1^H NMR (400 MHz, DMSO), *δ* (ppm): 2.40 (d, 3H), 3.86 (s, 3H), 6.21 (q, 1H), 6.95 (d, 1H), 6.98 (q, 1H), 7.68 (d, *J* = 8.4 Hz, 1H).

^13^C NMR (100 MHz, DMSO), *δ* (ppm): 18.60, 56.37, 101.17, 111.58, 112.58, 113.57, 126.93, 153.93, 155.24, 160.63, 162.84. ESI-MS *m*/*z*: 190; Anal. Calcd for C_11_H_10_O_3_: C: 69.47, H: 5.26. Found: C: 69.76, H: 5.48.

*7-Amino-4-methyl-2H-chromen-2-one (* ***6a***). This compound was prepared (147 mg, 84%) and identified according to the described procedure from Ethyl acetoacetate (130 mg, 1 mmol) and m-hydroxy aniline (109 mg, 1 mmol).

IR (*υ*_max_/cm^−1^): 1052, 1238, 1570, 1688, 3012, 3312, 3468.

^1^H NMR (400 MHz, CDCl_3_), (*δ*: ppm): 2.34 (d, 3H), 6.13 (q, 1H), 6.63 (s, 1H), 6.65 (d, *J* = 8.7 Hz, 1H), 7.50 (d, *J* = 8.7 Hz, 1H).

^13^C NMR (100 MHz, CDCl_3_), (*δ*: ppm): 19.40, 101.57, 109.40, 111.58, 114.57, 123.46, 153.17, 154.41, 154.63, 161.54. ESI-MS *m*/*z*: 175; Anal. Calcd for C_10_H_9_O_2_N: C: 68.57, H: 5.14, N: 8.02. Found: C: 68.79, H: 5.36, N: 8.21.

*7,8-Dihydroxy-4-methyl-2H-chromen-2-one (* ***7a***). This compound was obtained (169 mg, 88%) and identified according to the described procedure from Ethyl acetoacetate (130 mg, 1 mmol) and pyrogallol (126 mg, 1 mmol).

IR (*υ*_max_/cm^−1^): 629, 807, 1006, 1064, 1186, 1337, 1480, 1524, 1653, 2925, 3217.

^1^H NMR (400 MHz, DMSO), *δ* (ppm): 2.35 (d, 3H), 6.12 (q, 1H), 6.80 (d, *J* = 8.4 Hz, 1H), 7.08 (d, *J* = 8.8 Hz, 1H), 9.67 (s, 1H), 10.04 (s, 1H).

^13^C NMR (100 MHz, DMSO), *δ* (ppm): 18.72, 110.64, 112.56, 113.21, 115.95, 132.61, 143.74, 149.87, 154.41, 160.68. ESI-MS *m*/*z*: 192; Anal. Calcd for C_10_H_8_O_4_: C: 62.50, H: 4.17. Found: C: 62.86, H: 4.31.

*5,7-Dimethoxy-4-methyl-2h-chromen-2-one (* ***8a***). This compound was obtained (209 mg, 95%) and characterized according to the mentioned procedure from Ethyl acetoacetate (130 mg, 1 mmol) and 3,5-methoxy phenol (154 mg, 1 mmol).

IR (*υ*_max_/cm^−1^): 1130, 1353, 1460, 1616, 1733, 2939, 2992, 3024.

^1^H NMR (400 MHz, DMSO), *δ* (ppm): 2.46 (d, 3H), 3.83 (s, 3H), 3.84 (s, 3H), 5.98 (q, *J* = 1.2 Hz, 1H), 6.46 (d, *J* = 2.4 Hz, 1H), 6.54 (d, *J* = 2.4 Hz, 1H).

^13^C NMR (100 MHz, DMSO), *δ* (ppm): 24.05, 56.31, 56.65, 94.01, 95.99, 104.35, 111.08, 154.63, 156.79, 159.43, 160.15, 163.14. ESI-MS *m*/*z*: 220; Anal. Calcd for C_12_H_12_O_4_: C: 64.45, H: 5.45. Found: C: 64.22, H: 5.29.

*4-Methyl-2H-benzo[h]chromen-2-one (* ***9a***). This compound was prepared (193.2 mg, 92%) and identified according to the mentioned procedure from Ethyl acetoacetate (130 mg, 1 mmol) and *α*-naphthol (144 mg, 1 mmol).

IR (*υ*_max_/cm^−1^): 1044, 1275, 1572, 1750, 2922, 3012.

^1^H NMR (400 MHz, DMSO), *δ* (ppm): 2.53 (d, 3H), 6.50 (q, 1H), 7.68–7.74 (m, 2H), 7.78 (d, *J* = 8.8 Hz, 1H), 7.87 (d, *J* = 8.8 Hz, 1H), 8.02–8.06 (m, 1H), 8.34–8.37 (m, 1H).

^13^C NMR (100 MHz, DMSO), *δ* (ppm): 19.14, 114.34, 115.58, 121.74, 122.10, 122.66, 124.44, 127.86, 128.44, 129.12, 134.83, 150.13, 154.70, 160.13. ESI-MS *m*/*z*: 210; Anal. Calcd for C_14_H_10_O_2_: C: 80.00, H: 4.76. Found: C: 80.32, H: 4.94.

*4-Methyl-2H-benzo[f]chromen-2-one (* ***10a***). This compound was obtained (63 mg, 30%) and identified according to the described procedure from Ethyl acetoacetate (130 mg, 1 mmol) and *β*-naphtol (144 mg, 1 mmol).

IR (*υ*_max_/cm^−1^): 1044, 1215, 1514, 1630, 3052.

^1^H NMR (400 MHz, CDCl_3_), *δ* (ppm): 2.45 (d, 3H), 6.32 (q, 1H), 7.32–7.60 (m, 4H), 7.9 (d, *J* = 9.0 Hz, 1H), 8.54 (d, *J* = 9.0 Hz, 1H).

^13^C NMR (100 MHz, CDCl_3_), *δ* (ppm): 24.40, 109.47, 115.70, 117.76, 123.60, 126.35, 126.53, 128.91, 129.84, 134.58, 151.40, 154.96, 160.70. ESI-MS *m*/*z*: 210; Anal. Calcd for C_14_H_10_O_2_: C: 80.00, H: 4.76. Found: C: 80.45, H: 4.89.

*4,6,8-Trimethyl-2h-chromen-2-one (* ***11a***). Reaction of Ethyl acetoacetate (130 mg, 1 mmol) and 2,4-xylenol (122 mg, 1 mmol), according to the described procedure, did not yield the mentioned product.

## 3. Result and Discussion

Due to environmental concerns, in the last decade a variety of catalysts has been introduced in organic synthesis; among them are organocatalysts which have shown recyclability and nontoxicity as well as efficiency in producing key products. One of the most interesting supramolecular organic structures that have played so many roles in different areas especially in chemistry is calixarene and its derivatives.

**Scheme 2:**
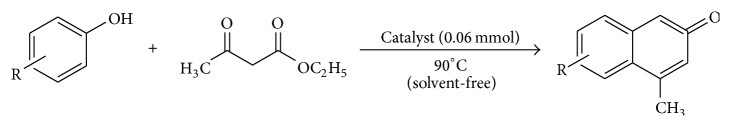
General procedure for preparation of coumarin derivatives with a variety of phenols using calix[4]arene sulfonic acid.

In the recent years, calix[4]arene tetrasulfonic acid as an organic catalyst has shown considerable capability in organic reactions and has been used as a novel efficient acidic organocatalyst in esterification [53, 54], Mannich type reaction [55, 56], and synthesis of xanthones, dixanthones [57], and bis(indolyl)methanes [58]. In this paper we aimed to report a green recognized procedure for the one-pot construction of coumarin derivatives using nonhazardous and recyclable organocatalyst, calix[4]arene sulfonic acid, under solvent-free condition. To survey the applicability of the catalyst for synthesis of pharmaceutically interesting compounds, a variety of coumarins were prepared via direct coupling of phenols and ethylacetoacetate ester in the presence of a catalytic amount (0.06 mmol) of the organocatalyst (Scheme 2).

The product structures and their corresponding melting points are shown in Table 1. As can be seen, the results clearly indicated good-to-high yields for coumarin derivatives except three compounds which were obtained in small quantities. To produce coumarin derivatives, mechanism of the reaction proceeds similarly to previously prepared xanthone derivatives through direct in situ cyclization of intermediates [57]. Exploring the outcome of the cyclization reaction between phenols and Ethyl acetoacetate as well as overall yields pointed out that some functional groups on phenolic substrate accelerate the ring closure and promote the product yields obviously.

As can be deduced from Table 1, enhancing the electron density will cause higher yields. Yields were promoted by the presence of electron releasing groups such as amino, hydroxyl, and methoxy groups (**5a**–**9a**). As was expected, phenols with electron donating groups such as hydroxy and methoxy activate the phenolic moieties to react faster with Ethyl acetoacetate, and consequently the products were obtained in higher yields and shorter times.

To find the best condition and optimize the reaction conditions especially from the environmental point of view, performing some experiments for possible modification on the reaction was necessary. So, we studied the reaction parameters including solvent, amount of catalyst, and reaction temperature. Reaction of resorcinol and Ethyl acetoacetate (**2a**) was considered as a typical reaction and the reaction parameters were changed and the yields were monitored consequently. At first, reaction medium was investigated using polar and nonpolar solvents such as tetrahydrofuran (THF), dichloromethane (DCM), n-hexane, acetonitrile, and solvent-free condition. Temperature and amount of catalyst were unchanged through the reactions. The result indicated that solvent-free condition gave the higher amount of the product in shorter periods (Table 2). However, in addition to higher yields, employing solvent-free protocol reduces the environmental pollutions and hazardous organic solvents. To reach the optimum catalytic activity of the catalyst, the different amounts of the catalyst were evaluated with some try-outs. So, according to Table 2, some experiments were done and the minimum amount of the catalyst with the acceptable result was considered as the most favorable amount of the catalyst. Determination of the optimum reaction temperature, which is critical for energy consumption issues, was conducted with some experiments in the range of 25 to 120°C. Results indicate that higher temperatures are needed to provide sufficient energy for nucleophilic attack by phenolic rings and consequently ring closure. Temperatures above 90°C have shown excellent results in the least time (Table 2).

**Figure 4:**
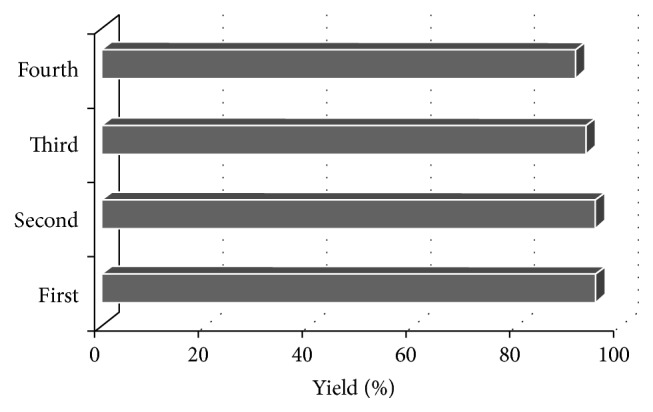
Efficiency of the calixarene sulfonic acid (CSA) in the synthesis of coumarins.

To explore the potential applicability of this notable organocatalyst in catalytic organic reactions especially in producing fine chemicals as well as to cover industrial concern in reducing the chemical pollutants in an environment, reusability of the catalyst has been practiced and the results were acceptable. Hence, after each reaction, catalyst was recovered by washing the precipitate with specified amounts of deionized water to dissolve the organocatalyst. Then, on filtration, aqueous solution of catalyst was evaporated, dried, and adjusted for in further successive reactions. The results showed that the recycled catalyst was still as efficient as the fresh one even after four runs of usage, and decreases in product yields are negligible, Figure 4.

## 4. Conclusions

In this research, potential applicability of* p*-sulfonic acid calix[4]arene (CSA) as a catalyst in the synthesis of pharmaceutically significant coumarin derivatives was evaluated. Efficient catalytic activity as well as recyclability makes CSA an exciting organocatalyst for researchers who are searching for more environmentally friendly catalysts with less harmful and more proficient capabilities. Moreover, herein, the earlier strategies in the synthesis of coumarin derivatives were developed using CSA as a catalyst in one-pot and solvent-free procedure. In this study we demonstrated especial and valuable organocatalyst for the synthesis of coumarins.

## Figures and Tables

**Table 1:** Coumarins prepared from tetrasulfonic acid calixarene via Pechmann reaction.

Substrate	Product	Time (h)	Yield (%)	M. P. (found)	M. P. (obtained)
1	**1a**	24	15	79–81 [46]	78-79
2	**2a**	2.5	95	182–184 [47]	183–185
3	**3a**	4	75	241-242 [46]	240–242
4	**4a**	2.5	50	130-131 [48]	132
8	**5a**	2	90	158–160 [47]	158-159
6	**6a**	2.5	84	220–224 [49]	222–224
7	**7a**	2.5	88	234-235 [47]	232–234
8	**8a**	1.5	95	—	173–175
9	**9a**	4	92	153–155 [47]	151–154
10	**10a**	10	30	182-183 [50]	180-181
11	**11a**	24	—	—	—

M. P.: melting point.

**Table 2:** Optimization of the reaction medium for the synthesis of 4-methyl-2H-chromen-2-one (1)^a^.

Entry	Catalyst (mg)	Solvent	Temp. (°C)	Time (h)	Yield (%)^b^
1	25	THF	80	5	60
2	25	CH_3_CN	80	5	67
3	25	CH_2_Cl_2_	80	12	30
4	25	n-Hexane	80	12	20
5	25	Solvent-free	80	3	78
6	15	Solvent-free	80	5	50
7	25	Solvent-free	80	3	78
8	45	Solvent-free	80	3	88
9	60	Solvent-free	80	2.5	90
10	45	Solvent-free	r.t	24	30
11	45	Solvent-free	50	10	60
12	45	Solvent-free	80	3	88
***13***	***45***	***Solvent-free***	***90***	***2.5***	***95***
14	45	Solvent-free	120	2.5	96

^a^Reaction condition: resorcinol (1 mmol) with Ethyl acetoacetate (1 mmol), *p*-sulfonic acid calix[4]arene.

^b^Isolated yield.

## References

[1] Sharma R., Arya V. J. (2011). A review on fruits having anti-diabetic potential. *Journal of Chemical and Pharmaceutical Research*.

[2] Murrey R. D. H., Medez D., Brown S. A. (1982). *The Natural Coumarins Occurrences, Chemistry and Biochemistry*.

[3] Paramjeet K. M., Dipak S. H., Arti D. J. (2012). Comparative study of microwave and conventional synthesis and pharmacological activity of coumarins: a review. *Journal of Chemical and Pharmaceutical Research*.

[4] Singer L. A., Kong N. P. (1966). Vinyl radicals; Stereoselectivity in hydrogen atom transfer to equilibrated isomeric vinyl radicals. *Journal of American Chemical Society*.

[5] Lake B. G. (1999). Coumarin metabolism, toxicity and carcinogenicity: relevance for human risk assessment. *Food and Chemical Toxicology*.

[6] Çakmak H. M., Kahraman S., Bayansal F., Çetinkaya S. (2012). A novel study on ZnO nanostructures: coumarin effect. *Philosophical Magazine Letters*.

[7] Zahradnik M. (1992). *The Production and Application of Fluorescent Brightening Agents*.

[8] Weigt S., Huebler N., Strecker R., Braunbeck T., Broschard T. H. (2012). Developmental effects of coumarin and the anticoagulant coumarin derivative warfarin on zebrafish (*Danio rerio*) embryos. *Reproductive Toxicology*.

[9] Çamur M., Bulut M., Kandaz M., Güney O. (2009). Effects of coumarin substituents on the photophysical properties of newly synthesised phthalocyanine derivatives. *Supramolecular Chemistry*.

[10] Chen J., Liu W., Ma J. (2012). Synthesis and properties of fluorescence dyes: tetracyclic pyrazolo [3,4-*b*] pyridine-based coumarin chromophores with intramolecular charge transfer character. *The Journal of Organic Chemistry*.

[11] Rohini K., Srikumar P. S. (2014). Therapeutic role of coumarins and coumarin-related compounds. *Journal of Thermodynamics & Catalysis*.

[12] Lacy A., O'Kennedy R. (2004). Studies on coumarins and coumarin-related compounds to determine their therapeutic role in the treatment of cancer. *Current Pharmaceutical Design*.

[13] Yang X.-W., Xu B., Ran F.-X., Wang R.-Q., Wu J., Cui J.-R. (2007). Inhibitory effects of 11 coumarin compounds against growth of human bladder carcinoma cell line E-J in vitro. *Journal of Chinese Integrative Medicine*.

[14] Hamdi N., Bouabdallah F., Romerosa A., Benhassen R. (2010). Expedious synthesis for *α*, *β*-unsaturated coumarin derivatives using boran chelates: a novel class of potential antibacterial and antioxidant agents. *Comptes Rendus Chimie*.

[15] Kadhum A. A. H., Al-Amiery A. A., Musa A. Y., Mohamad A. B. (2011). The antioxidant activity of new coumarin derivatives. *International Journal of Molecular Sciences*.

[16] Smitha G., Sanjeeva R., Smitha G. (2004). ZrCl_4_-catalyzed Pechmann reaction: synthesis of coumarins under solvent-free conditions. *Synthetic Communications*.

[17] Kotali A., Lafazanis I. S., Harris P. A. (2008). Synthesis of 6,7-diacylcoumarins via the transformation of a hydroxy into a carbonyl group. *Synthetic Communications*.

[18] Kostova I. P., Manolov I. I., Nicolova I. N., Danchev N. D. (2001). New metal complexes of 4-methyl-7-hydroxycoumarin sodium salt and their pharmacological activity. *II Farmaco*.

[19] O'Kennedy R., Thornes R. D. (1997). *Coumarins: Biology, Applications and Mode of Action*.

[20] Nofal Z. M., El-Zahar M. I., Abd El-Karim S. S. (2000). Novel coumarin derivatives with expected biological activity. *Molecules*.

[21] Sahoo S. S., Shukla S., Nandy S., Sahoo H. B. (2012). Synthesis of novel coumarin derivatives and its biological evaluations. *European Journal of Experimental Biology*.

[22] Perkin W. H. (1868). XXIII.—On the hydride of aceto-salicyl. *Journal of Chemical Society*.

[23] Pechmann V. H., Duisberg C. (1884). Neue Bildungsweise der Cumarine. Synthese des Daphnetins. *Chemische Berichte*.

[24] Jones G. (1967). The Knoevenagel condensation. *Organic Reactions*.

[25] Brufola G., Fringuelli F., Piermatti O., Pizzo F. (1996). Simple and efficient one-pot preparation of 3-substituted coumarins in water. *Heterocycles*.

[26] Shirner R. L. (1942). The reformatsky reaction. *Organic Reactions*.

[27] Shah D. N., Shah N. M. (1955). The Kostanecki-Robinson acylation of 5-hydroxy-6-acetyl-4-methylcoumarin. *Journal of the American Chemical Society*.

[28] Narasimhan N. S., Mali R. S., Barve M. V. (1979). Synthetic application of lithiation peactions; part XIII. Synthesis of 3-phenylcoumarins and their benzo derivatives. *Synthesis*.

[29] Yavari I., Hekmat-Shoar R., Zonouzi A. (1998). A new and efficient route to 4-carboxymethylcoumarins mediated by vinyltriphenylphosphonium salt. *Tetrahedron Letters*.

[30] Miros F. N., Huang G., Zhao Y., Sakai N., Matile S. (2015). Coumarin synthesis on *π*-acidic surfaces. *Supramolecular Chemistry*.

[31] Rahmatpour A., Mohammadian S. (2013). Polystyrene-supported TiCl_4_ as a novel, efficient and reusable polymeric Lewis acid catalyst for the chemoselective synthesis and deprotection of 1,1-diacetates under eco-friendly conditions. *Comptes Rendus Chimie*.

[32] Albadi J., Shirini F., Abasi J., Armand N., Motaharizadeh T. (2013). A green, efficient and recyclable poly (4-vinylpyridine)-supported copper iodide catalyst for the synthesis of coumarin derivatives under solvent-free conditions. *Comptes Rendus Chimie*.

[33] Castanheiro R. A. P., Silva A. M. S., Campos N. A. N., Nascimento M. S. J., Pinto M. M. M. (2009). Antitumor activity of some prenylated xanthones. *Pharmaceuticals*.

[34] Yang W., De Villiers M. M. (2004). The solubilization of the poorly water soluble drug nifedipine by water soluble 4-sulphonic calix[*n*]arenes. *European Journal of Pharmaceutics and Biopharmaceutics*.

[35] Yang W., Otto D. P., Liebenberg W., de Villiers M. M. (2008). Effect of para-sulfonato-calix[n]arenes on the solubility, chemical stability, and bioavailability of a water insoluble drug nifedipine. *Current Drug Discovery Technologies*.

[36] Yang W., de Villiers M. M. (2004). Aqueous solubilization of furosemide by supramolecular complexation with 4-sulphonic calix[n]arenes. *Journal of Pharmacy and Pharmacology*.

[37] Yang W., de Villiers M. M. (2005). Effect of 4-sulphonato-calix[n]arenes and cyclodextrins on the solubilization of niclosamide, a poorly water soluble anthelmintic. *The AAPS Journal*.

[38] Panchal J. G., Patel R. V., Menon S. K. (2010). Preparation and physicochemical characterization of carbamazepine (CBMZ): para-sulfonated calix[n]arene inclusion complexes. *Journal of Inclusion Phenomena and Macrocyclic Chemistry*.

[39] Wang G.-S., Zhang H.-Y., Ding F., Liu Y. (2011). Preparation and characterization of inclusion complexes of topotecan with sulfonatocalixarene. *Journal of Inclusion Phenomena and Macrocyclic Chemistry*.

[40] Paclet M.-H., Rousseau C. F., Yannick C., Morel F., Coleman A. W. (2006). An absence of non-specific immune response towards *para*-sulphonato-calix[*n*] arenes. *Journal of Inclusion Phenomena and Macrocyclic Chemistry*.

[41] Yang W., Manek R., Kolling W. M. (2005). Physicochemical characterization of hydrated 4-sulphonato-calix [n] arenes: thermal, structural, and sorption properties. *Supramolecular Chemistry*.

[42] Lakouraj M. M., Tajbakhsh M., Tashakkorian H., Ghodrati K. (2007). Fast and efficient oxidation of sulfides to sulfones with N,N′-dibenzyl-N,N,N′,N′-tetramethyl diammonium permanganate. *Phosphorus, Sulfur and Silicon and the Related Elements*.

[43] Lakouraj M. M., Tajbakhsh M., Tashakkorian H. (2007). Montmorillonite K10 catalyzed selective oxidation of sulfides to sulfoxides using hydrogen peroxide. *Letters in Organic Chemistry*.

[44] Lakouraj M. M., Tajbakhsh M., Tashakkorian H. (2007). Ion exchange resin catalyzed selective oxidation of sulfides to sulfoxides using hydrogen peroxide. *Monatshefte für Chemie*.

[45] Baghbanian S. M., Rezaei N., Tashakkorian H. (2013). Nanozeolite clinoptilolite as a highly efficient heterogeneous catalyst for the synthesis of various 2-amino-4*H*-chromene derivatives in aqueous media. *Green Chemistry*.

[46] Atkins R. L., Bliss D. E. (1978). Substituted coumarins and azacoumarins. Synthesis and fluorescent properties. *Journal of Organic Chemistry*.

[47] Anjaeyulu A. S. R., Row L. R., Krishna C. S., Srinivasulu C. (1968). Synthesis of benzochromenes and related compounds I.5-6 and 7-8 benzochromanones and benzocoumarines. *Current Science*.

[48] Dong X. M., Revol J.-F., Gray D. G. (1998). Effect of microcrystallite preparation conditions on the formation of colloid crystals of cellulose. *Cellulose*.

[49] Keim W., Korth W., Wasserscheid P.

[50] Gui J., Cong X., Liu D., Zhang X., Hu Z., Sun Z. (2004). Novel Brønsted acidic ionic liquid as efficient and reusable catalyst system for esterification. *Catalysis Communications*.

[51] Gutsche C. D. (1998). *Calixarenes Revisited*.

[52] Shinkai S., Araki K., Tsubaki T., Some T., Manabe O. (1987). New syntheses of calixarene-*p*-sulphonates and *p*-nitrocalixarenes. *Journal of the Chemical Society, Perkin Transactions 1*.

[53] Fernandes S. A., Natalino R., Gazolla P. A. R., da Silva M. J., Jham G. N. (2012). p-Sulfonic acid calix[n]arenes as homogeneous and recyclable organocatalysts for esterification reactions. *Tetrahedron Letters*.

[54] Natalino R., Varejão E. V. V., da Silva M. J., Cardoso A. L., Fernandes S. A. (2014). p-Sulfonic acid calix[*n*]arenes: the most active and water tolerant organocatalysts in esterification reactions. *Catalysis Science & Technology*.

[55] da Silva D. L., Fernandes S. A., Sabino A. A., de Fátima Â. (2011). *p*-Sulfonic acid calixarenes as efficient and reusable organocatalysts for the synthesis of 3,4-dihydropyrimidin-2(1*H*)-ones/-thiones. *Tetrahedron Letters*.

[56] Shimizu S., Shimada N., Sasaki Y. (2006). Mannich-type reactions in water using anionic water-soluble calixarenes as recoverable and reusable catalysts. *Green Chemistry*.

[57] Lakouraj M. M., Tashakkorian H., Rouhi M. (2013). One-pot synthesis of xanthones and dixanthones using calix [4] arene sulfonic acid under solvent free condition. *Chemical Science Transactions*.

[58] Baghbanian S. M., Babajani Y., Tashakorian H., Khaksar S., Farhang M. (2013). P-sulfonic acid calix[4]arene: an efficient reusable organocatalyst for the synthesis of bis(indolyl)methanes derivatives in water and under solvent-free conditions. *Comptes Rendus Chimie*.

